# Sulindac, 3,3’-diindolylmethane and curcumin reduce carcinogenesis in the Pirc rat, an *Apc*-driven model of colon carcinogenesis

**DOI:** 10.1186/s12885-015-1627-9

**Published:** 2015-09-03

**Authors:** Angelo Pietro Femia, Paulo Victoria Soares, Cristina Luceri, Maura Lodovici, Augusto Giannini, Giovanna Caderni

**Affiliations:** 1 NEUROFARBA Department, Section of Pharmacology and Toxicology, University of Florence, 6 Viale Pieraccini, 50139 Florence, Italy; 2 Department of Pathology and Legal Medicine, Faculty of Medicine of Ribeirão Preto, University of São Paulo, São Paulo, Brazil; 3 Department of Pathology, General Hospital of Prato, Prato, Italy

## Abstract

**Background:**

Recently, we showed that Sulindac (SU; 320 ppm) reduces precancerous lesions in the colon of Pirc rats, mutated in the *Apc* gene*.* Surprisingly, previous data in *Apc*-mutated mice showed that SU, with reported efficacy in Familial Adenomatous Polyposis (FAP), increases colon carcinogenesis. Therefore, we assessed the effect of SU 320 ppm in a long-term carcinogenesis experiment in Pirc rats. Moreover, since side effects of SU hamper its chronic use and a combination of drugs could be more effective and less toxic than single agents, we also studied whether two natural compounds, 3,3’-diindolylmethane (DIM; 250 ppm) and curcumin (CUR; 2000 ppm), with or without lower doses of SU could affect carcinogenesis

**Methods:**

Pirc rats were fed an AIN76 diet containing SU, DIM and CUR and sacrificed at 8 months of age to measure intestinal tumours. Apoptosis and proliferation in the normal colon mucosa, as well as gene expression profile were studied

**Results:**

Colon tumours were significantly reduced by SU 320 ppm (62 % reduction over Controls), by DIM and CUR without or with SU 80 and 160 ppm (50, 53 and 58 % reduction, respectively) but not by SU 80 ppm alone. Total tumours (colon and small intestine) were reduced by SU (80 and 320 ppm) and by DIM and CUR. Apoptosis in the normal mucosa was significantly increased by SU 320 ppm, and slightly increased by DIM and CUR with or without SU. A slight reduction in *Survivin-Birc5* expression was observed with all the treatments compared to Controls. Proliferative activity was not varied

**Conclusions:**

The results on SU reinforce the validity of Pirc rats to identify chemopreventive products. Moreover, the efficacy of the DIM and CUR combination to lower colon tumours, suggests an alternative strategy to be exploited in patients at risk.

**Electronic supplementary material:**

The online version of this article (doi:10.1186/s12885-015-1627-9) contains supplementary material, which is available to authorized users.

## Background

The administration of drugs or natural compounds to prevent or slow down the process of colon carcinogenesis (chemoprevention), has been suggested to lower cancer risk in patients with familial adenomatous polyposis (FAP) or individuals with a personal history of sporadic colorectal cancer (CRC) [1–4]. However, significant side effects associated with the use of non-steroidal anti-inflammatory drugs (NSAID) like Sulindac (SU) or Celecoxib, two of the most effective chemopreventive drugs, have argued against their chronic use and evidenced the need for alternative regimens devoid of toxicity, but still able to lower carcinogenesis [1]. Indeed, to identify such compounds, one has to rely on adequate experimental models. Since *Apc* gene mutations are a key event in colon carcinogenesis, rodent models carrying germline mutations in this gene have been developed and widely used [5, 6]. Pirc rats (Polyposis in the rat colon) mutated in the *Apc* gene (F344/NTac-Apc ^am1137^) were described a few years ago [7, 8]. At variance with established genetic models like *Apc*^Min^ (Min) mice, developing tumours mostly in the small intestine [5, 6], Pirc rats develop tumours also in the colon, and are thus potentially a more appropriate model for CRC and FAP [7, 8]. Recently, we reported that Sulindac (SU, 320 ppm in the diet) reduces precancerous lesions in the Pirc rat colon [9]. This result, while suggesting that Pirc rats might be useful to identify chemopreventive drugs, needs to be assessed in a long-term carcinogenesis experiment. Intriguingly, the protective effect of SU in Pirc rats contrasts with previous reports in *Apc*-mutated mice, where similar dosages of SU decrease small intestinal carcinogenesis but increase colon tumours [10, 11].

Curcumin (CUR), an active molecule from *Curcuma longa*, shows chemopreventive activity in various experimental settings, and acts, at least in part, to increase apoptosis [12–17] and to down-regulate Cyclooxygenase-2 (COX-2), which is overexpressed in colon carcinogenesis [18]. 3,3’-diindolylmethane (DIM), a derivative of indole-3-carbinol present in cruciferous plants, has been reported to lower colon inflammation and tumorigenesis in mice [19, 20]. Moreover DIM has been shown to synergize with apoptosis inducers, down-regulating the anti-apoptotic protein Survin (Birc5), over-expressed in *Apc*-mutated cells [20, 21]. Recently, we have shown that the apparently normal colon mucosa (NM) of Pirc rats over-expresses *Birc5* [9], suggesting that a combination of DIM with an apoptosis inducer like CUR may reduce colon carcinogenesis in these *Apc*-mutated rats. Accordingly, experimental studies have shown that a combination of drugs is often more effective than individual agents [2, 3].

Based on these premises, the aims of this long-term carcinogenesis experiment in Pirc rats were: 1) to assess the effect of 320 ppm of SU, the same dosage decreasing colon precancerous lesions [9]; 2) to study the chemopreventive activity of a combination of DIM (250 ppm) and CUR (2000 ppm); 3) to study the chemopreventive activity of a combination of DIM (250 ppm) and CUR (2000 ppm) with low doses of SU (80 ppm or 160 ppm); 4) to test a lower dose of SU (80 ppm) alone.

Since SU, CUR and DIM may affect apoptosis and proliferation in the NM and tumours, we studied these parameters. Moreover, we determined the expression of some genes related to carcinogenesis (*Birc5, Casp7, Casp3, Bax, Bcl2, Cox2*) in the NM. Oxidant/pro-oxidant activity, potentially affecting cancer risk, was studied as well [13, 22].

## Methods

### Animals

Pirc (F344/NTac-Apc ^am1137^) rats were obtained from Taconic (Taconic Farms, Inc. USA) and bred in CESAL (University of Florence, Italy) in accordance with the Italian Guidelines for Animal Care, DL 116/92, application of the European Communities Council Directive (86/609/EEC). The Pirc colony was maintained by mating heterozygous Pirc rats with wild type Fisher F344/NTac rats (Taconic Farms, Inc. USA); pups were genotyped at one month of age according to Amos-Landgraf and colleagues [7]. Rats were maintained in polyethylene cages with wire tops and bottoms and maintained at a temperature of 22 °C, with a 12:12-h light–dark cycle, under an experimental protocol approved by the Institutional Animal Care and Use Committee (IACUC) of the University of Florence and performed according to the Italian Law on Animal Welfare (DL 116/92). Detailed description of experimental procedures was done in accordance with the ARRIVE guidelines and could be found in Additional file 1: Table S1

### Diet composition and treatments of the animals

Components for the preparation of the AIN76 diet were purchased from Piccioni (Gessate, Milan, Italy). SU, DIM and CUR were purchased from Sigma-Aldrich (Milan, Italy).

For the long-term carcinogenesis experiment, male Pirc rats, aged 6 weeks, were randomly allocated into: Controls: fed a standard diet (AIN76 diet) (*n* = 9); DIM CUR SU 80 group: treated with the same control diet containing SU (80 ppm in the diet), DIM (250 ppm in the diet according to Bhatnagar and colleagues [20]) and CUR (2000 ppm as previously reported [17]) (*n* = 5); DIM CUR SU 160 group: treated with the diet containing SU (160 ppm), DIM (250 ppm) and CUR (2000 ppm) (*n* = 6); SU 320 group: treated with the diet containing SU (320 ppm) (*n* = 4); DIM CUR group: treated with DIM (250 ppm) and CUR (2000 ppm) (*n* = 5); SU 80 group: treated with the diet containing SU (80 ppm) (*n* = 5).

Assuming for rats a mean body weight of 300 g and 15 g of daily diet consumption, a diet containing 320 ppm SU provides 4.8 mg of SU per rat/day, i.e. 16 mg/Kg body weight. Making the proportion for a man of 70 kg, this value corresponds to 1120 mg of SU/day, three times higher than the highest dose of SU recommended for chemoprevention (400 mg/d) [23]. However, taking into account the different metabolic rate in humans and rats [24], and the fact that 320 ppm is the dose with strong chemopreventive activity in rats [25], we assumed that 320 ppm, 160 ppm and 80 ppm of SU can be roughly compared to high, medium and low equivalent doses, respectively, of SU used for chemoprevention in FAP [23–26].

For carcinogenesis experiments, treatments were continued up to 8 months of age, and during this time animals were monitored for body weight and possible rectal bleeding. For the experiments on the effect of SU 320 ppm on apoptosis, animals (8 controls and 8 SU-treated) were sacrificed at 2 and 5 months of age (*n* = 4 in both groups). Animals were sacrificed by CO_2_ asphyxia.

### Sample collection and tumour analysis

At sacrifice the entire intestine (from pylorus to anus) was dissected and flushed with cold saline. Starting from the duodenum, segments of the small intestine (6 cm long) were longitudinally cut and spread on filter paper with the mucosa surface upward to assess tumours as described [9]. Similarly, the entire colon was longitudinally opened and macroscopic tumours were assessed as described [9]. Tumours were assessed by a researcher (G.C.) who was unaware of the experimental codes.

Tumours were enumerated and for each rat a mean tumour volume was calculated. To do this, we assumed the tumour as a sphere with a mean diameter that was evaluated at sacrifice. After this determination, a segment of apparently morphologically normal mucosa (NM) of the colon, as well as samples of selected tumours, were dissected and processed for histology in order to study proliferative activity and apoptosis as described below. Sections stained with haematoxylin and eosin were also used to determine the type of tumours by histopathological examination, performed by a pathologist (A.G.) unaware of the experimental codes. Tumours were evaluated on the basis of the histotype, grading and pattern of growth, using the same criteria adopted in human pathology [27, 28]. Accordingly, all the tumours were classified as adenomas with high or low grade of dysplasia. High grade of dysplasia: is defined as a lesion showing a moderate-severe grade of dysplasia corresponding to a carcinoma *in-situ* or intramucosal carcinoma; low grade of dysplasia: is defined as a lesion showing mild grade dysplasia.

### Determination of proliferation and apoptosis in the intestinal mucosa and tumours

Proliferative activity in the apparently morphologically normal mucosa (NM) was evaluated by determining Proliferating Cell Nuclear Antigen (PCNA) immunoreactivity in longitudinal sections of NM, using a mouse monoclonal antibody (PC-10, Santa Cruz, CA, USA) [29]. Proliferative activity was evaluated by a single observer on coded samples, in at least 15 full longitudinal crypt sections of the NM and expressed as labelling index (LI): the number of cells positive to PCNA/cells scored × 100 [29].

Apoptosis was evaluated in coded histological longitudinal sections (4 μm thick) of the NM and selected samples of tumours stained with haematoxylin-eosin, as reported [29]. An apoptotic index (AI) was determined, that is, the number of apoptosis/cells scored ×100; we scored at least 1000 cells. In the normal mucosa (NM), apoptosis was expressed as apoptosis/crypt and at least 15 full longitudinal crypt sections were scored.

### Semi-quantitative RT-PCR and oxidant/antioxidant status in the plasma

Gene expression was evaluated in the apparently normal colon mucosa (NM), taken at the sacrifice as described [9]. The primers used for the amplification of the different genes have been previously described [9]. For each target gene, the relative amount of mRNA in the samples was calculated as the ratio of each gene to β-actin mRNA [9].

The determination of Reactive Oxygen Species (ROS), ferric reducing ability of plasma (FRAP) and carbonyl residues (CO) in the plasma, was carried out as previously described [9].

### Statistical evaluation of the data

Data obtained from individual rats in the different experimental groups were summarised for quantitative continuous responses by calculating group means and standard deviations. Comparisons among the different groups were analysed with one-way ANOVA, post-hoc comparisons were analysed with Bonferroni’s test for multiple comparison performed with Graph-pad (p-level fixed at 0.05, two-sided). The effect of SU 320 ppm on apoptosis was also evaluated in two separate short-term treatment experiments (1 and 4 months of treatment). In this case, we used two-way ANOVA to evaluate the effect of Sulindac and, at the same time, that of the different experiment.

## Results

The mean weight of the rats at the beginning of the long-term carcinogenesis experiment (6 weeks of age) was 180 ± 8 g (means ± SE, *n* = 34). At sacrifice, when the animals were 8 months old, the mean weight was similar among dietary groups, with no apparent signs of toxicity from the treatments (445 ± 15 g, 461 ± 14 g, 420 ± 18 g, 456 ± 10 g, 429 ± 13 g and 406 ± 2 g; means ± SE, in Controls, DIM CUR SU 80, DIM CUR SU 160, SU 320, DIM CUR and SU 80 groups, respectively).

**Fig. 1:**
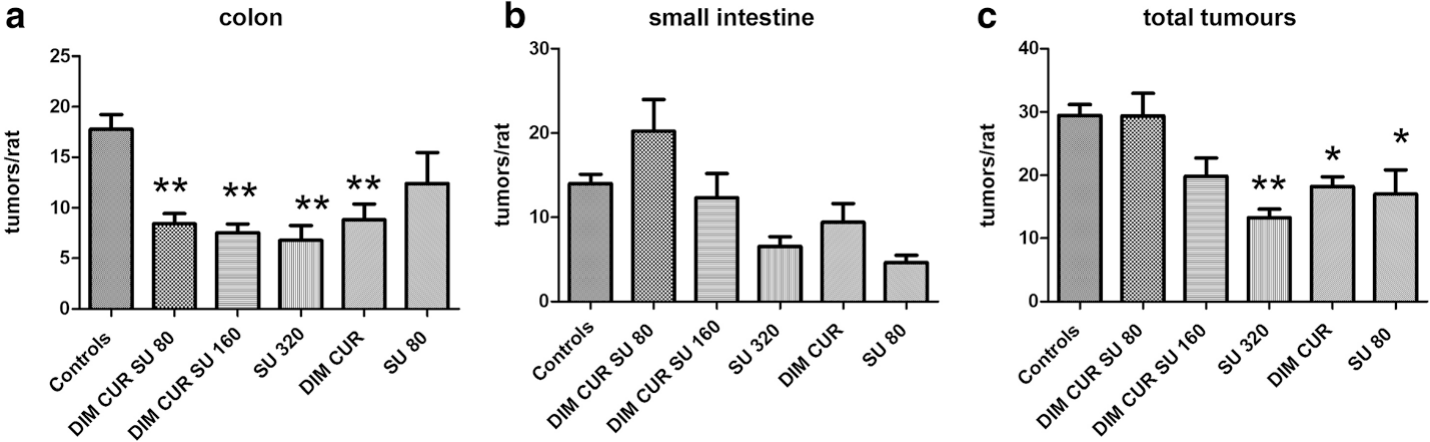
Mean number of tumours/rat in the colon (panel **a**), in the small intestine (panel **b**) and in the entire intestine (colon and small intestine together) (panel **c**), in the various experimental groups. Bars represent mean values + SE. The number of rats per group were: 9 in Controls, 5 in DIM CUR SU 80, 6 in DIM CUR SU 160, 4 in SU 320, 5 in DIM CUR and 5 in SU 80 groups. **, *: significantly different (*P* < 0.01, *P* < 0.05, respectively) compared with Controls

The determination of tumours showed that in all the experimental groups (Fig. 1, panel a), with the exception of the SU 80 ppm group, the number of tumours in the colon was significantly lower than in Controls. The volumes of these tumours were similar among groups (data not shown).

Regarding the small intestine, none of the treatments significantly varied the number of tumours in the small intestine (Fig. 1, panel b). However, in the experimental groups the volumes of the small intestinal tumours were slightly smaller than in the Control group (mean volumes (mm^3^) were: 52.4 ± 10.0 [9], 25.4 ± 10.1 [5], 20.0 ± 4.5 [6], 19.5 ± 5.1 [4], 22.4 ± 4.1 [5] and 28.2 ± 6.0 [5] in Controls, DIM CUR SU 80, DIM CUR SU 160, SU 320, DIM CUR and SU 80 groups, respectively; (number of rats in each group)), and the DIM CUR SU 160 was significantly lower (*P* < 0.05) than the Control group.

Considering the total number of tumours (i.e. both colonic and small intestinal tumours), a significant lower number of tumours was observed in the groups treated with 320 ppm and 80 ppm of SU as well in the DIM CUR group (Fig. 1, panel c).

**Fig. 2:**
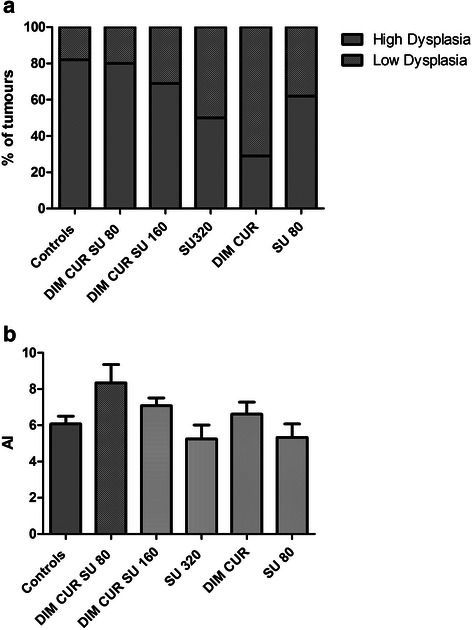
Panel **a**: percent of tumours in the various experimental groups showing high or low dysplasia. Panel **b**: Apoptotic index (AI) in the tumours of the various groups (in both panels: *n* = 17, 10, 13, 10, 7, 8, in Controls, DIM CUR SU 80, DIM CUR SU 160, SU 320, DIM CUR and SU 80 groups, respectively)

In a subset of colon tumours (*n* = 17, 10, 13, 10, 7, 8, in Controls, DIM CUR SU 80, DIM CUR SU 160, SU 320, DIM CUR and SU 80 groups, respectively) we determined the histology and the level of apoptosis (Fig. 2). All the tumours were classified as adenomas; interestingly, the percent of highly dysplastic adenomas tends to be lower in the CUR DIM and SU 320 compared to Controls (Fig. 2, panel a). Apoptosis in the same subset of tumours was similar among the groups (Fig. 2, panel b).

**Fig. 3:**
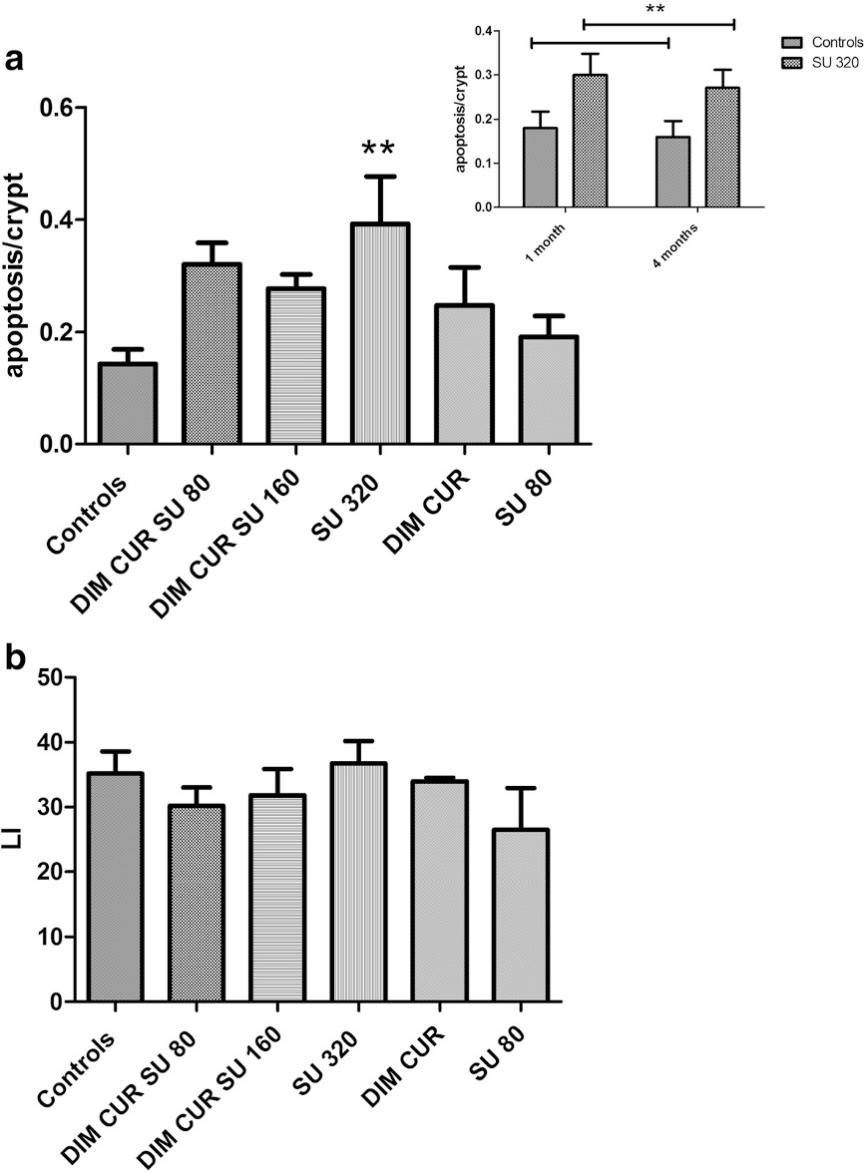
Panel **a**: apoptosis/crypt in the apparently normal mucosa (NM) of the various experimental groups. Bars represent mean + SE. *n* = 8, 5, 6, 4, 5, 5 in Controls, DIM CUR SU 80, DIM CUR SU 160, SU 320, DIM CUR and SU 80 groups, respectively. **: significantly different (*P* < 0.01) when compared with Controls. Insert of panel **a**: apoptosis/crypt in the NM of rats treated with a Control diet or with the same diet supplemented with SU 320 ppm for 1 or 4 months. Bars represent mean numbers + SE. *n* = 4 in each group. **: significantly different (*P* < 0.05) compared with Controls (with two-way analysis of variance for the effect of Sulindac). Panel **b**: proliferative activity, expressed as LI (labelling index) in the NM of the different groups. Bars are means + SE. *n* = 7, 5, 5, 4, 3, 4 in Controls, DIM CUR SU 80, DIM CUR SU 160, SU 320, DIM CUR and SU 80 groups, respectively

We also measured apoptosis in the NM and found that the highest dose of SU (320 ppm) caused a significant increase in apoptosis compared with Controls (Fig. 3, panel a). The groups treated with DIM CUR with or without the combination of SU, also showed a number of apoptotic cells slightly higher than Controls, but the effect was not statistically significant (Fig. 3, panel a).

The effect of the high dose of SU (320 ppm) on apoptosis was then confirmed in Pirc rats treated with the same dose for a shorter period (1 or 4 months), a time sufficient to decrease the number of precancerous lesions in the colon [9]. Indeed, with these short-term administrations as well, rats treated with SU showed a significantly higher apoptosis than Controls (Fig. 3, insert in panel a). Proliferative activity in the NM was similar among groups (Fig. 3 panel b).

**Fig. 4:**
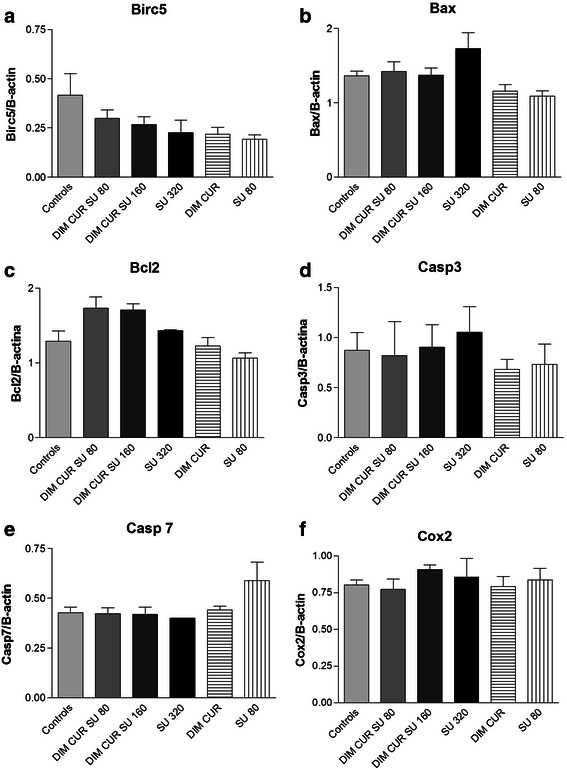
Relative expression of Birc5, Bax, Bcl2,Casp3, Casp7, Cox2 (panels **a**, **b**, **c**, **d**, **e** and **f**, respectively) in the colon mucosa of the variousexperimental groups. Bars represent means ± SE, *n* =8, 5, 6, 3, 5 and 5 in Controls, DIM CUR SU 80, DIM CUR SU 160, SU 320, DIM CUR and SU 80 groups, respectively

In order to understand the molecular mechanisms of action underlying the protective effects observed in the colon, we studied the expression of some genes affecting apoptosis and tumour development in the apparently normal colon mucosa (NM). *Birc5* (*Survivin*, Fig. 4, panel a) expression was slightly, although not significantly, lower in treated groups compared with Controls. Similarly, the expression of other apoptosis-related genes (*Bax, Bcl2, Casp 3 and Casp7*) was not varied (Fig. 4, panels b, c, d and e). *Cox2* gene expression was also similar among groups (Fig. 4, panel f).

Plasma FRAP did not vary significantly among groups (108 ± 8 μM [5], 140 ± 27 μM [5], 167 ± 25 μM [6], 121 ± 10 μM [3], 152 ± 43 μM [5] and 192 ± 61 μM [5] in Controls, DIM CUR SU 80, DIM CUR SU 160, SU 320, DIM CUR and SU 80 groups, respectively, means ± SE (n)). Similarly, ROS and CO did not vary (data not shown).

## Discussion

SU was the first NSAID to show chemopreventive activity in FAP patients [4]. Several studies have thereafter confirmed its ability to reduce the number of adenomas in the colon and rectum [1, 4]. Despite the fact that FAP patients on SU may still develop adenocarcinomas, chronic treatment with this drug has been suggested as an option to postpone surgery [1, 4].

SU treatment has also been demonstrated to decrease carcinogenesis in carcinogen-induced models of colon cancer [25]. Surprisingly, studies in Min mice, showed that SU increased carcinogenesis in the colon, raising perplexities on the validity of that genetic model to identify chemopreventive drugs for colon cancer [10, 11, 30]. Recently, we reported that 320 ppm of SU in the diet decreases precancerous lesions in the colon of Pirc rats [9]. The results of the present study, obtained in a long-term carcinogenesis study, showed that SU 320 ppm decreased colon and total tumors. Therefore, while confirming our previous results on preneoplastic lesions, these results indicate that indeed the Pirc rat may be a valid model to identify chemopreventive agents.

We also found that 80 ppm of SU tends to lower carcinogenesis in both the colon and small intestine, giving a statistically significant reduction only with respect to the total number of tumours (Fig. 1, panel c). A similar slight effect of this SU dose has been reported in carcinogen-induced rats [31], suggesting, together with the results of clinical studies in FAP, that higher doses of SU are indeed necessary to afford a full chemopreventive effect in the colon [1, 32].

Although SU has not been associated with serious cardiovascular effects such as those caused by COX-2 selective inhibitors, chronic use of this drug at relatively high doses can expose the patients to gastrointestinal toxicity [1, 23]. Accordingly, in the present study we were also interested in studying the chemopreventive activity of a combination of DIM (250 ppm) and CUR (2000 ppm) with or without lower doses of SU (80 ppm or 160 ppm).

Regarding CUR and DIM given together without SU, we observed that this combination was able to lower carcinogenesis in the colon, and, although not significantly, also in the small intestine. On the contrary, when DIM and CUR were administered together with 160 or 80 ppm of SU, we observed that while in the colon these combinations showed a protective effect, this effect was not observed in the small intestine.

Therefore, taken together, our results show that addition of SU to CUR and DIM did not afford further protection compared to the combination of CUR and DIM on its own.

It is also interesting to note that regarding colon tumours, and the total number of tumours, the chemopreventive activity of the combination of DIM and CUR is comparable to the effect afforded by 320 ppm of SU. Therefore, long-term treatment with a combination of CUR and DIM could be envisaged to prevent tumours in FAP patients. In fact, a combination of CUR and Quercetin administered for six months has been reported to be tolerated and effective in reducing adenomas in FAP, although side-effects possibly emerging after longer periods are not known [33]. Since chemoprevention in FAP requires long treatments, a possible strategy to reduce potential toxicity without lowering the chemopreventive effectiveness could be to alternate SU with a combination of CUR and DIM.

Regarding the mechanism of action, we found that at least SU 320 ppm, significantly increased apoptosis in the NM, in line with previous data in FAP patients [34]. We also observed that even a shorter period of treatment with SU 320 ppm (one or four months) was able to increase apoptosis in the NM, further reinforcing the data observed in the long-term carcinogenesis experiment.

Regarding CUR and DIM, previous studies have shown that CUR increases apoptosis in the small intestinal mucosa of Min mice [14, 16], while DIM may synergize with apoptosis inducers like butyrate through down-regulation of the anti-apoptotic molecule survivin [20]. We observed that all the experimental diets caused a slight down-regulation of the anti-apoptotic gene S*urvivin (Birc5),* which is up-regulated in Pirc rats compared to wt rats [9], while we did not find differences in the expression of other apoptosis related genes. Only a slight increase in apoptosis was observed in the groups treated with diets containing DIM and CUR. Given the efficacy of these experimental diets to lower colon carcinogenesis, although we can speculate that their chemopreventive effect may be due to an increase in apoptosis in NM, additional mechanisms of action, to be further investigated, are probably involved.

Since higher proliferative activity in the colon mucosa has been associated with an increased risk of developing cancer [35], we measured this parameter in the NM. Proliferative activity was not affected by the dietary treatments. Regarding SU, this result is in line with previous studies in FAP patients in which SU did not cause a variation in proliferative activity in the NM [34, 36]. As for CUR and DIM, both have been shown to arrest cell proliferation of cancer cells *in vitro* [15, 37], while *in vivo* studies showed an increase in cell proliferation in Min mice treated with CUR 1000 ppm [14]. To our knowledge, no previous studies evaluated the effect of DIM on the proliferative activity of the colon.

Due to the large number of colon tumours present in the Pirc rats, it was not possible to study the histology and the level of apoptosis in each tumour. Therefore, we selected a subset of tumours from each dietary group, which were shown to be adenomas of various grades of dysplasia. Interestingly, although the volume of this subset of tumours was similar, the percent of highly dysplastic adenomas tended to be lower in the CUR DIM and SU 320 compared to Controls. On the contrary, despite previous reports indicating a pro-apoptotic effects of SU, CUR and DIM also in tumour cells [20, 38, 39], we did not observe variation in apoptosis.

## Conclusions

In conclusion, we showed that colon tumours in Pirc rats were significantly reduced by SU 320 ppm and by DIM and CUR with or without SU 80 and 160 ppm. The efficacy of the DIM and CUR combination to lower colon tumours in this relevant model of colon cancer, suggests an alternative strategy to be exploited in patients at risk.

## Additional file

Additional file 1: Table S1. Detailed description of experimental procedures in accordance with the ARRIVE guidelines (Kilkenny et al., 2010) (DOCX 24 kb)
