# Supplementary material for: Sulindac, 3,3’-diindolylmethane and curcumin reduce carcinogenesis in the Pirc rat, an Apc-driven model of colon carcinogenesis
Source: BMC Cancer. 2015 Sep 3;15:611. doi: 10.1186/s12885-015-1627-9 (PMC4559292; doi:10.1186/s12885-015-1627-9)
Supplement: Additional file 1: Table S1. — Detailed description of experimental procedures in accordance with the ARRIVE guidelines (Kilkenny et al., 2010) (DOCX 24 kb) [file 12885_2015_1627_MOESM1_ESM.docx]

Additional file 1: Table S1. Detailed description of experimental procedures in accordance with the ARRIVE guidelines (Kilkenny et al., 2010).

|  | **ITEM** | **ARRIVE guidelines RECOMMENDATION** | **Information given in the paper** |
| --- | --- | --- | --- |
| Title | 1 | Provide as accurate and concise a description of the content of the article as possible | Sulindac, 3,3’-diindolylmethane and curcumin reduce carcinogenesis in the Pirc rat, an Apc-driven model of colon carcinogenesis |
| Abstract | 2 | Provide an accurate summary of the background, research objectives, including details of the species or strain of animal used, key methods, principal findings and conclusions of the study. | **Background.** Recently, we showed that Sulindac (SU; 320 ppm) reduces precancerous lesions in the colon of Pirc rats, mutated in the Apc gene. Surprisingly, previous data in Apc-mutated mice showed that SU, with reported efficacy in Familial Adenomatous Polyposis (FAP), increases colon carcinogenesis. Therefore, we assessed the effect of SU 320 ppm in a long-term carcinogenesis experiment in Pirc rats. Moreover, since side effects of SU hamper its chronic use and a combination of drugs could be more effective and less toxic than single agents, we also studied whether two natural compounds, 3,3’-diindolylmethane (DIM; 250 ppm) and curcumin (CUR; 2000 ppm), with or without lower doses of SU could affect carcinogenesis. Methods. Pirc rats were fed an AIN76 diet containing SU, DIM and CUR and sacrificed at 8 months of age to measure intestinal tumours. Apoptosis and proliferation in the normal colon mucosa, as well as gene expression profile were studied. Results. Colon tumours were significantly reduced by SU 320 ppm (62% reduction over Controls), by DIM and CUR without or with SU 80 and 160 ppm (50%, 53% and 58% reduction, respectively) but not by SU 80 ppm alone. Total tumours (colon and small intestine) were reduced by SU (80 and 320 ppm) and by DIM and CUR. Apoptosis in the normal mucosa was significantly increased by SU 320 ppm, and slightly increased by DIM and CUR with or without SU. A slight reduction in Survivin-Birc5 expression was observed with all the treatments compared to Controls. Proliferative activity was not varied. Conclusions. The results on SU reinforce the validity of Pirc rats to identify chemopreventive products. Moreover, the efficacy of the DIM and CUR combination to lower colon tumours, suggests an alternative strategy to be exploited in patients at risk. |
| INTRODUCTION |  |  |  |
| Background | 3a | Include sufficient scientific background (including relevant references to previous work) to understand the motivation and context for the study,  and explain the experimental approach and rationale. | The administration of drugs or natural compounds to prevent or slow down the process of colon carcinogenesis (chemoprevention), has been suggested to lower cancer risk in patients with familial adenomatous polyposis (FAP) or individuals with a personal history of sporadic colorectal cancer (CRC) (1-4). However, significant side effects associated with the use of non-steroidal anti-inflammatory drugs (NSAID) like Sulindac (SU) or Celecoxib, two of the most effective chemopreventive drugs, have argued against their chronic use and evidenced the need for alternative regimens devoid of toxicity, but still able to lower carcinogenesis (1). Indeed, to identify such compounds, one has to rely on adequate experimental models. Since Apc gene mutations are a key event in colon carcinogenesis, rodent models carrying germline mutations in this gene have been developed and widely used (5-6). |
|  | 3b | Explain how and why the animal species and model being used can address the scientific objectives and, where appropriate, the study’s relevance to human biology. | Pirc rats (Polyposis in the rat colon) mutated in the Apc gene (F344/NTac-Apc am1137) were described a few years ago (7-8). At variance with established genetic models like ApcMin (Min) mice, developing tumours mostly in the small intestine (5-6), Pirc rats develop tumours also in the colon, and are thus potentially a more appropriate model for CRC and FAP (7-8). Recently, we reported that Sulindac (SU, 320 ppm in the diet) reduces precancerous lesions in the Pirc rat colon (9). This result, while suggesting that Pirc rats might be useful to identify chemopreventive drugs, needs to be assessed in a long-term carcinogenesis experiment. Intriguingly, the protective effect of SU in Pirc rats contrasts with previous reports in Apc-mutated mice, where similar dosages of SU decrease small intestinal carcinogenesis but increase colon tumours (10-11). |
| Objectives | 4 | Clearly describe the primary and any secondary objectives of the study, or specific hypotheses being tested. | Based on the above premises, the aims of this long-term carcinogenesis experiment in Pirc rats were: 1) to assess the effect of 320 ppm of SU, the same dosage decreasing colon precancerous lesions (9); 2) to study the chemopreventive activity of a combination of DIM (250 ppm) and CUR (2000 ppm); 3) to study the chemopreventive activity of a combination of DIM (250 ppm) and CUR (2000 ppm) with low doses of SU (80 ppm or 160 ppm); 4) to test a lower dose of SU (80 ppm) alone. Since SU, CUR and DIM may affect apoptosis and proliferation in the NM and tumours, we studied these parameters. Moreover, we determined the expression of some genes related to carcinogenesis (Birc5, Casp7, Casp3, Bax, Bcl2, Cox2) in the NM. Oxidant/pro-oxidant activity, potentially affecting cancer risk, was studied as well (13, 22). |
| METHODS |  |  |  |
| Ethical statement | 5 | Indicate the nature of the ethical review permissions, relevant licences (e.g.  Animal [Scientific Procedures] Act 1986), and national or institutional  guidelines for the care and use of animals, that cover the research. | Pirc (F344/NTac-Apc am1137) rats were obtained from Taconic (Taconic Farms, Inc. USA) and bred in CESAL (University of Florence, Italy) in accordance with the Italian Guidelines for Animal Care, DL 116/92, application of the European Communities Council Directive (86/609/EEC). The Pirc colony was maintained by mating heterozygous Pirc rats with wild type Fisher F344/NTac rats (Taconic Farms, Inc. USA); pups were genotyped at one month of age according to Amos-Landgraf and colleagues (7). Rats were maintained in polyethylene cages with wire tops and bottoms and maintained at a temperature of 22° C, with a 12:12-h light-dark cycle, under an experimental protocol approved by the Institutional Animal Care and Use Committee (IACUC) of the University of Florence and performed according to the Italian Law on Animal Welfare (DL 116/92). |
| Study design | 6 | For each experiment, give brief details of the study design including:  a. The number of experimental and control groups.  b. Any steps taken to minimise the effects of subjective bias when allocating animals to treatment (e.g. randomisation procedure) and when  assessing results (e.g. if done, describe who was blinded and when).  c. The experimental unit (e.g. a single animal, group or cage of animals). | For the long-term carcinogenesis experiment, male Pirc rats, aged 6 weeks, were randomly allocated into: Controls: fed a standard diet (AIN-76 diet) (n=9); DIM CUR SU 80 group: treated with the same control diet containing SU (80 ppm in the diet), DIM (250 ppm in the diet according to Bhatnagar and colleagues (20)) and CUR (2000 ppm as previously reported (17)) (n= 5); DIM CUR SU 160 group: treated with the diet containing SU (160 ppm), DIM (250 ppm) and CUR (2000 ppm) (n=6); SU 320 group: treated with the diet containing SU (320 ppm) (n= 4); DIM CUR group: treated with DIM (250 ppm) and CUR (2000 ppm) (n= 5); SU 80 group: treated with the diet containing SU (80 ppm) (n=5).  Tumours were assessed by a researcher (G.C.) who was unaware of the experimental codes. |
| Experimental  Procedures | 7 | For each experiment and each experimental group, including controls, provide precise details of all procedures carried out. For example:  a. How (e.g. drug formulation and dose, site and route of administration, anaesthesia and analgesia used [including monitoring], surgical  procedure, method of euthanasia). Provide details of any specialist equipment used, including supplier(s).  b. When (e.g. time of day).  c. Where (e.g. home cage, laboratory, water maze).  d. Why (e.g. rationale for choice of specific anaesthetic, route of administration, drug dose used). | Rats were maintained in polyethylene cages with wire tops and bottoms and maintained at a temperature of 22° C, with a 12:12-h light-dark cycle, under an experimental protocol approved by the Institutional Animal Care and Use Committee (IACUC) of the University of Florence and performed according to the Italian Law on Animal Welfare (DL 116/92).  Further details on each experiment are given in the Method section of the manuscript |
| Experimental  animals | 8 | a: Provide details of the animals used, including species, strain, sex, developmental stage (e.g. mean or median age plus age range) and  weight (e.g. mean or median weight plus weight range).  b. Provide further relevant information such as the source of animals, international strain nomenclature, genetic modification status (e.g.  knock-out or transgenic), genotype, health/immune status, drug or test  naïve, previous procedures, etc. | Pirc rats (Polyposis in the rat colon) mutated in the Apc gene (F344/NTac-Apc am1137) were described a few years ago (7-8). At variance with established genetic models like ApcMin (Min) mice, developing tumours mostly in the small intestine (5-6), Pirc rats develop tumours also in the colon, and are thus potentially a more appropriate model for CRC and FAP (7-8).  Pirc (F344/NTac-Apc am1137) rats were obtained from Taconic (Taconic Farms, Inc. USA) and bred in CESAL (University of Florence, Italy) in accordance with the Italian Guidelines for Animal Care, DL 116/92, application of the European Communities Council Directive (86/609/EEC).  For the long-term carcinogenesis experiment, male Pirc rats, aged 6 weeks, were randomly allocated into the various experimental groups (see Method section) |
| Housing and  husbandry | 9 | a. Housing (type of facility e.g. specific pathogen free [SPF]; type of cage or housing; bedding material; number of cage companions; tank shape and material etc. for fish).  b. Husbandry conditions (e.g. breeding programme, light/dark cycle,  temperature, quality of water etc for fish, type of food, access to food and water, environmental enrichment).  c. Welfare-related assessments and interventions that were carried out prior to, during, or after the experiment. | Pirc (F344/NTac-Apc am1137) rats were obtained from Taconic (Taconic Farms, Inc. USA) and bred in CESAL (University of Florence, Italy) in accordance with the Italian Guidelines for Animal Care, DL 116/92, application of the European Communities Council Directive (86/609/EEC). The Pirc colony was maintained by mating heterozygous Pirc rats with wild type Fisher F344/NTac rats (Taconic Farms, Inc. USA); pups were genotyped at one month of age according to Amos-Landgraf and colleagues (7).  Rats were maintained in polyethylene cages with wire tops and bottoms and maintained at a temperature of 22° C, with a 12:12-h light-dark cycle, under an experimental protocol approved by the Institutional Animal Care and Use Committee (IACUC) of the University of Florence and performed according to the Italian Law on Animal Welfare (DL 116/92). |
| Sample size | 10 | a. Specify the total number of animals used in each experiment, and the  number of animals in each experimental group.  b. Explain how the number of animals was arrived at. Provide details of any  sample size calculation used.  c. Indicate the number of independent replications of each experiment, if  relevant. | For the long-term carcinogenesis experiment, male Pirc rats, aged 6 weeks, were randomly allocated into: Controls: fed a standard diet (AIN-76 diet) (n=9); DIM CUR SU 80 group: treated with the same control diet containing SU (80 ppm in the diet), DIM (250 ppm in the diet according to Bhatnagar and colleagues (20)) and CUR (2000 ppm as previously reported (17)) (n= 5); DIM CUR SU 160 group: treated with the diet containing SU (160 ppm), DIM (250 ppm) and CUR (2000 ppm) (n=6); SU 320 group: treated with the diet containing SU (320 ppm) (n= 4); DIM CUR group: treated with DIM (250 ppm) and CUR (2000 ppm) (n= 5); SU 80 group: treated with the diet containing SU (80 ppm) (n=5).  The number of rats per group was based on our previous study (9) in which we found a statistically significant reduction in the number of preneoplastic lesions (MDF) in rats treated with SU 320 ppm compared with Controls (n= 6 and 4 in Controls and SU, respectively). |
| Allocating  animals to  experimental  groups | 11 | a. Give full details of how animals were allocated to experimental groups,  including randomisation or matching if done.  b. Describe the order in which the animals in the different experimental  groups were treated and assessed | For the long-term carcinogenesis experiment, male Pirc rats, aged 6 weeks, were randomly allocated into the various experimental groups (see Method section).  The allocation into the various group was based on the availability of rats from our colony.  Pirc rats were randomly allocated into the different experimental groups, avoiding, whenever possible, to allocate brothers into the same experimental group. |
| Experimental  outcomes | 12 | Clearly define the primary and secondary experimental outcomes assessed (e.g. cell death, molecular markers, behavioural changes). | These have been described in the Method and Result sections |
| Statistical  methods | 13 | Provide details of the statistical methods used for each analysis.  b. Specify the unit of analysis for each dataset (e.g. single animal, group of  animals, single neuron).  c. Describe any methods used to assess whether the data met the assumptions of the statistical approach. | Data obtained from individual rats in the different experimental groups were summarised for quantitative continuous responses by calculating group means and standard deviations. Comparisons among the different groups were analysed with one-way ANOVA, post-hoc comparisons were analysed with Bonferroni’s test for multiple comparison performed with Graph-pad (p-level fixed at 0.05, two-sided). The effect of SU 320 ppm on apoptosis was also evaluated in two separate short-term treatment experiments (1 and 4 months of treatment). In this case, we used two-way ANOVA to evaluate the effect of Sulindac and, at the same time, that of the different experiment. |
| RESULTS |  |  |  |
| Baseline data | 14 | For each experimental group, report relevant characteristics and health status of animals (e.g. weight, microbiological status, and drug or test naïve)  prior to treatment or testing. (This information can often be tabulated). | The mean weight of the rats at the beginning of the long-term carcinogenesis experiment (6 weeks of age) was 180 ± 8 g (means ± SE, n=34). At sacrifice, when the animals were 8 months old, the mean weight was similar among dietary groups, with no apparent signs of toxicity from the treatments (445 ± 15 g, 461 ± 14 g, 420 ± 18 g, 456 ± 10 g, 429 ± 13 g and 406 ± 2 g; means ± SE, in Controls, DIM CUR SU 80, DIM CUR SU 160, SU 320, DIM CUR and SU 80 groups, respectively). |
| Numbers  analysed | 15 | a. Report the number of animals in each group included in each analysis.  Report absolute numbers (e.g. 10/20, not 50%).  b. If any animals or data were not included in the analysis, explain why. | See Result secion |
| Outcomes and  estimation | 16 | Report the results for each analysis carried out, with a measure of precision  (e.g. standard error or confidence interval). | See Result section |
| Adverse events | 17 | a. Give details of all important adverse events in each experimental group.  b. Describe any modifications to the experimental protocols made to  reduce adverse events. | No adverse effects were observed. |
| DISCUSSION |  |  |  |
| Interpretation/  scientific  implications | 18 | a. Interpret the results, taking into account the study objectives and  hypotheses, current theory and other relevant studies in the literature.  b. Comment on the study limitations including any potential sources of bias,  any limitations of the animal model, and the imprecision associated with the results.  c. Describe any implications of your experimental methods or findings for the replacement, refinement or reduction (the 3Rs) of the use of animals  in research. | See Discussion section |
| Generalisability/  translation | 19 | Comment on whether, and how, the findings of this study are likely to translate to other species or systems, including any relevance to human  biology. | We showed that colon tumours in Pirc rats were significantly reduced by SU 320 ppm and by DIM and CUR with or without SU 80 and 160 ppm. The efficacy of the DIM and CUR combination to lower colon tumours in this relevant model of colon cancer, suggests an alternative strategy to be exploited in patients at risk. |
| Funding | 20 | List all funding sources (including grant number) and the role of the funder(s) in the study. | This work has received financial support from the Istituto Toscano Tumori (ITT) and by the University of Florence (Fondo ex-60%). |

References:

1. Kilkenny C, Browne WJ, Cuthill IC, Emerson M, Altman DG (2010) Improving Bioscience Research Reporting: The ARRIVE Guidelines for Reporting Animal Research. PLoS Biol 8(6): e1000412. doi:10.1371/journal.pbio.1000412
